# RETRACTION: Oxaliplatin Activates P53/miR‐34a/Survivin Axis in Inhibiting the Progression of Gastric Cancer Cells

**DOI:** 10.1002/iid3.70191

**Published:** 2025-04-11

## Abstract

**RETRACTION**: Q. Guo, X.‐Y. Wang, Y.‐C. Zhai, Y.‐W. Dong, and Q.‐S. He, “Oxaliplatin Activates P53/miR‐34a/Survivin Axis in Inhibiting the Progression of Gastric Cancer Cells,” *Immunity, Inflammation and Disease* 12, no. 9 (2024): e70004, https://doi.org/10.1002/iid3.70004.

The above article, published online on 10 September 2024 in Wiley Online Library (wileyonlinelibrary.com), has been retracted by agreement between the journal Editor‐in‐Chief, Marc Veldhoen; and John Wiley & Sons Ltd. The retraction has been agreed upon due to insufficient information included within the methodology, which prevents accurate reproduction of the study. Furthermore, there are concerns that the beta actin bands included in Figure 3a do not originate from the same gel as the other bands shown in this figure. Finally, the cell line used in this study was reported to be contaminated. The authors were contacted for comment and supporting data, but they did not respond. The editors consider the results and conclusions unreliable. The authors were informed of the retraction.

**RETRACTION**: Q. Guo, X.‐Y. Wang, Y.‐C. Zhai, Y.‐W. Dong, and Q.‐S. He, “Oxaliplatin Activates P53/miR‐34a/Survivin Axis in Inhibiting the Progression of Gastric Cancer Cells,” *Immunity, Inflammation and Disease* 12, no. 9 (2024): e70004, https://doi.org/10.1002/iid3.70004.

The above article, published online on 10 September 2024 in Wiley Online Library (wileyonlinelibrary.com), has been retracted by agreement between the journal Editor‐in‐Chief, Marc Veldhoen; and John Wiley & Sons Ltd. The retraction has been agreed upon due to insufficient information included within the methodology, which prevents accurate reproduction of the study. Furthermore, there are concerns that the beta actin bands included in Figure 3a do not originate from the same gel as the other bands shown in this figure. Finally, the cell line used in this study was reported to be contaminated. The authors were contacted for comment and supporting data, but they did not respond. The editors consider the results and conclusions unreliable. The authors were informed of the retraction.

